# Supercomplexity: bridging the gap between aesthetics and cognition

**DOI:** 10.3389/fnins.2025.1552363

**Published:** 2025-07-29

**Authors:** Alessandro Bortolotti, Giulia Candeloro, Riccardo Palumbo, Pier Luigi Sacco

**Affiliations:** ^1^Department of Neuroscience, Imaging and Clinical Sciences, “G. d’Annunzio” University of Chieti-Pescara, Chieti, Italy; ^2^metaLAB (at) Harvard, Cambridge, MA, United States

**Keywords:** network neuroscience, expertise, predictive processing, neural plasticity, supercomplexity

## Abstract

This study presents a cognitive neuroscience framework for understanding what we term “supercomplex experiences,” a concept describing experiences that simultaneously engage multiple neural networks and cognitive faculties in ways that resist decomposition into simpler processes. Drawing on recent advances in network neuroscience, we argue that these experiences emerge from the coordinated activity of distributed brain systems, including the salience network, default mode network, and central executive network. These experiences are distinguished by five essential characteristics: (1) simultaneous engagement of multiple neural networks, (2) specialized neural architectures developed through training, (3) specialized conceptual frameworks and vocabularies, (4) emergent properties from dynamic interactions, and (5) coherent gestalt properties. Through examination of expert performance in domains such as wine tasting, musical performance, visual art, perfumery, and several others we reveal how these experiences are characterized by sophisticated integration of sensory, emotional, and cognitive processes, implemented through dynamic network interactions and expertise-dependent neural plasticity. Our framework emphasizes three key mechanisms underlying supercomplex experiences: predictive processing architectures that generate and update multi-level predictions, expertise-dependent network reorganization that enables enhanced sensory discrimination and conceptual integration, and dynamic network flexibility that supports adaptive processing of complex stimuli. While acknowledging debates between different theoretical approaches, we show how interoceptive predictions and embodied simulations, implemented through the anterior insula and related networks, provide a foundation for integrating bodily signals with external sensory input. The development of expertise in domains characterized by supercomplex experiences involves significant modifications of neural architecture, from local circuit refinement to large-scale network reorganization. This work extends beyond existing frameworks in cognitive neuroscience by providing a mechanistic account of how the brain processes and generates richly textured, multifaceted experiences that have previously been studied primarily through separate disciplinary lenses. The framework has implications for understanding expertise development, individual differences in complex skill acquisition, and the neural bases of sophisticated cognitive-perceptual capabilities.

## Introduction

Consider these four brief texts:

“Earthy aromas of bark, sand, truffles, graphite, rust, terracotta and sandalwood rise up from the deep with Burgundian poise; fruitier orange zest and preserved lemons glimmer in their wake, while riper incense and tar lurk in this dense forest of aromas. Physics itself gives way as the line between reduction and oxidation is blurred. There’s such richness to the center palate, but the fruit there is vivacious and elegant, swaddled in robust but generous tannins that breach the outermost reaches of the mouth. The finish is hauntingly long, the after effects of the acidity unworldly. This is certainly in a quiet, reserved stage of evolution, and will need time to fully mature in bottle.”
“A concentrated red, with intense flavors of black cherry, plum, violet, stony mineral and wild herbs. Ample flesh covers the dense matrix of tannins, but this retains plenty of structure and should develop well. Offers a lingering, salty aftertaste.”
“The [omitted] takes a slightly different read of the vintage, with drier and more austere fruit flavors. I get some tannic astringency as well on the finish with a point of bitterness. Compared to many of its peers that produced soft and richly opulent wines in the warm 2015 vintage, [omitted]’s expression is a bit more angular and edgy. You get dark fruit tones with sour cherry and spice on the close. One thing it does share in common is that balsamic aromatic intensity that you encounter so readily in [omitted].”
“The [omitted] is dark and intense in its aromatics, displaying a mix of earthy mineral and soil tones, offset by crushed black cherry, savory herbs, leather, and spice. On the palate, soft, fleshy textures usher in ripe cherry fruits, offset by a mix of brisk acids and saturating minerals, as hints of exotic spice buzz upon the senses, leaving a coating of fine tannin in their wake. The finish is long and structured, flexing its hulky tannins, as only a hint of dried black cherry, minerals, and spice linger. The [omitted] needs time and lots of it, but I believe its primary fruit and acids will 1 day tame the massive structure that is currently dominating the wine’s personality.”

What do they all have in common, apart from clearly referring to a wine-tasting experience? These reviews describe the same wine, the Renieri 2015 Brunello di Montalcino, assessed by four different professional wine critics^1^. Each critic encounters the identical physical stimulus yet produces markedly different descriptive accounts, and ultimately “experiences.” This pattern exemplifies what we term a “supercomplex experience” that simultaneously engages multiple cognitive, perceptual, and affective systems in ways that resist decomposition into simpler processes. While sensory input from the wine remains constant across tasters, each critic constructs a distinct experiential world through their encounter with it, drawing on specialized perceptual capacities, conceptual frameworks, and expressive vocabularies developed through years of expertise. This raises fundamental questions about the nature of perception, expertise, and the integration of sensory and cognitive processes in certain experiences.

This study presents a cognitive neuroscience framework for understanding supercomplex experiences, drawing on recent advances in network neuroscience, predictive processing, and the philosophy of perception and expertise. We argue that supercomplex experiences emerge from the coordinated activity of distributed brain systems and are characterized by five essential features: (1) simultaneous engagement of multiple neural networks, (2) specialized neural architectures developed through training, (3) specialized conceptual frameworks and vocabularies, (4) emergent properties from dynamic interactions, and (5) coherent gestalt properties.

Through examination of domains such as wine tasting, musical performance, visual art, and other areas of expertise, we reveal how these experiences are characterized by sophisticated integration of sensory, emotional, and cognitive processes. This integration is implemented through dynamic network interactions and expertise-dependent neural plasticity that enable increasingly refined perceptual discrimination and conceptual categorization.

Our framework emphasizes three key mechanisms underlying supercomplex experiences: predictive processing architectures that generate and update multi-level predictions, allowing experts to detect and categorize subtle differences that are imperceptible to novices; an expertise-dependent network reorganization that enables enhanced sensory discrimination and conceptual integration through neural plasticity; and dynamic network flexibility that supports adaptive processing of complex stimuli through metastable patterns of coordination between large-scale brain networks.

This work extends beyond existing frameworks in cognitive neuroscience by providing a mechanistic account of experiences that have previously been studied primarily through separate disciplinary lenses. By bringing together insights from network neuroscience, expertise studies, and the philosophy of perception, we offer a unified theoretical approach to understanding some of the richest and most complex forms of human experience.

The framework has implications for understanding expertise development, individual differences in complex skills acquisition, and the neural bases of sophisticated cognitive-perceptual capabilities. It also offers insights into how humans navigate complex domains that exceed the capacity of purely analytical approaches, suggesting that supercomplex experiences represent a distinct and important mode of cognitive engagement with the world.

The paper proceeds as follows. We begin with the neurophilosophical foundations of our framework, examining how insights from Dewey’s pragmatism, predictive processing, and enactivism converge to illuminate the nature of integrated experience (Gallagher and Allen, 2018). After defining supercomplex experiences through five essential criteria and distinguishing them from merely complex phenomena through boundary cases, we survey key domains where these experiences manifest. Our unified theoretical framework then integrates embodiment, interoception, and network dynamics to explain how these experiences emerge. The subsequent sections examine the neural implementation of supercomplexity, beginning with the fundamental network dynamics that enable integrated processing, followed by analysis of how these networks support real-time experience and the development of expertise through exemplification and neural plasticity. We then explore two crucial dimensions of supercomplex experiences: the sophisticated cross-modal integration that characterizes expert perception and communication, and the neural synchronization that occurs during collective experiences. The discussion section considers theoretical implications, methodological challenges, and future directions, while our examination of practical applications demonstrates the framework’s relevance across educational, clinical, and technological domains. Throughout, we argue that supercomplex experiences represent a distinct mode of human cognitive engagement that cannot be reduced to simpler components, requiring instead an understanding of how multiple neural systems coordinate to create emergent experiential wholes that define expertise in its most developed forms.

## The neurophilosophical foundations of supercomplex experiences

The concept of supercomplex experiences that we develop in this paper finds important philosophical antecedents in John Dewey’s theory of experience, particularly as elaborated in *Art* as Experience (1934). Dewey’s careful distinction between ordinary experience and what he calls “*an* experience” provides crucial conceptual foundations for understanding how experiences can simultaneously engage multiple dimensions of human cognition while maintaining a coherent unity. However, contemporary cognitive neuroscience allows us to extend and enrich Dewey’s insights in ways that illuminate the neural mechanisms supporting such integrated experiences.

For Dewey, “an experience” occurs when various elements and meanings are integrated into a unified whole that maintains its own individuating quality while flowing from part to part toward its fulfillment: “we have *an* experience when the material experienced runs its course to fulfillment. Then and then only it is integrated within and demarcated in the general stream of experience from other experiences. Such an experience is a whole and carries with it its own individualizing quality and self-sufficiency. It is *an* experience” (p. 35, emphasis original).

This conceptualization bears interesting similarities to what we term supercomplex experiences. Both share a quality of integration that resists decomposition into simpler elements, both involve the emergence of a coherent whole with distinctive phenomenological properties, and both emphasize a dynamic process that unfolds over time rather than a static state. These parallels are not accidental but reflect a deep affinity between Dewey’s phenomenological analysis and our neuroscientific account.

Dewey’s insight that genuine experiences involve “doing and undergoing” in rhythmic alternation helps explain why supercomplex experiences often require both active engagement and receptive attention. The wine critic must actively explore the wine’s properties—swirling, sniffing, tasting—while also remaining receptively attentive to the subtle qualities that emerge. This rhythmic alternation between active exploration and receptive attention resonates with our emphasis on the dynamic interaction between bottom-up sensory processing and top-down predictive models in expert perception (Basso et al., 2021). The wine critic’s perceptual exploration is guided by top-down predictions about what features to attend to, while remaining open to bottom-up signals that may violate or refine these predictions.

Particularly significant is Dewey’s insistence that in genuine experiences, practical, emotional, and intellectual properties are integrated phases of a single dynamic whole rather than separate elements. As he writes, “the aesthetic quality that rounds out an experience into completeness and unity is emotional,” pointing toward the intrinsic role of affect in organizing complex experiential wholes (Bortolotti et al., 2024). This philosophical insight aligns with contemporary neuroscientific research on emotional integration (Damasio and Carvalho, 2013) and the role of the salience network in coordinating between cognitive and affective processing. The salience network, anchored in the anterior insula and anterior cingulate cortex, integrates affective signals with cognitive processes, providing a neural substrate for the emotional-cognitive integration that Dewey described phenomenologically.

Dewey’s account provides a valuable philosophical groundwork to our framework of supercomplex experiences, which extends his insights in several important ways. Where Dewey could only speculate about the mechanisms supporting integrated experiences, our framework identifies specific neural systems and their coordinated activity as the substrate for supercomplex experiences. We propose that the dynamic interaction between the default mode network, salience network, and central executive network provides the neural basis for the integration that Dewey described phenomenologically (Menon and D’Esposito, 2022). This identification of specific neural mechanisms does not reduce Dewey’s phenomenological insights to mere neural activity but rather enriches them by identifying the physical processes that enable the phenomenological unity he described.

Contemporary cognitive science offers multiple theoretical perspectives that bear on our understanding of supercomplex experiences. The Free Energy Principle (FEP) developed by Friston (2010) provides one influential framework, proposing that brain function can be understood in terms of minimizing prediction error or “free energy.” While this computational approach might initially seem distant from Dewey’s pragmatism, recent developments suggest intriguing possibilities for integration. The FEP applies across multiple spatial and temporal scales, from cellular processes to social interactions, making it a potentially powerful framework for understanding how different levels of cognitive and sensorimotor processing might be integrated in supercomplex experiences.

Enactivist approaches to cognition, developing independently from pragmatism, share many of its commitments to embodied, action-oriented understanding. Enactivism views cognition not as the representation of a pre-given world by a pre-given mind but as the enactment of a world and mind through a history of embodied action (Thompson, 2007). This perspective resonates with Dewey’s rejection of the spectator theory of knowledge in favor of understanding cognition as fundamentally embedded in action. The concept of sense-making in enactivist literature (De Jaegher and Di Paolo, 2007) parallels Dewey’s account of how organisms actively create meaning through their transactions with the environment.

Recent integrative work has begun to explore how these different theoretical traditions might be reconciled. Bruineberg et al. (2018) have proposed understanding active inference (Friston, 2013), a key pillar of the FEP framework, not as constructing internal models of an external world but as attunement to environmental affordances (Friston et al., 2017). This interpretation brings predictive processing frameworks closer to ecological and enactivist perspectives. Similarly, Gallagher and Allen (2018) propose a “predictive engagement” framework that combines predictive processing computational principles with an enactivist emphasis on organism-environment coupling.

These theoretical convergences are particularly relevant for understanding supercomplex experiences. The skilled intentionality framework developed by Bruineberg et al. (2018) explains how experts develop heightened sensitivity to relevant affordances in their domain through the refinement of predictive models attuned to action possibilities. This integration helps explain how wine critics, musicians, and artists develop the sophisticated perceptual-motor skills that characterize expertise in supercomplex domains.

The multiscale perspective offered by contemporary theories also resonates with pragmatist insights about the continuity between different levels of organization. The concept of Markov blankets, i.e., statistical boundaries that separate systems from their environments while allowing for causal interaction, provides formal tools for understanding what Dewey described qualitatively as the transactional nature of experience. Ramstead et al. (2019) have extended this concept to show how cognitive boundaries exist at multiple nested scales, with different boundaries becoming relevant depending on the phenomenon being investigated.

This multiscale, integrative perspective has important implications for our understanding of supercomplex experiences. Rather than viewing neural processes as the sole determinants of experience, we can understand them as participating in broader patterns of organism-environment interaction. The sophisticated integration that characterizes supercomplex experiences emerges not from brain processes alone but from the dynamic coupling between neural activity, bodily states, environmental affordances, and cultural practices (Ramstead et al., 2016).

Vernazzani’s (2023) recent work on aesthetic perception provides another important bridge between philosophical and neuroscientific approaches. His analysis of how aesthetic experiences modify perception through processes of exemplification complements our account by illuminating how attention to aesthetic, expressive, or design properties in one context can scaffold the recognition of similar properties in other contexts. This process of exemplification depends on what Vernazzani terms “aesthetic looking”: the informed attention to aesthetic, expressive, or design properties. Our neural account provides a mechanistic basis for understanding how such aesthetic looking might be implemented through the coordination of attentional networks and expertise-dependent perceptual systems.

The integration of these diverse theoretical perspectives suggests that supercomplex experiences cannot be fully understood through any single framework. Instead, they require a pluralistic approach that recognizes how neural mechanisms, embodied action, environmental interaction, and cultural practices all contribute to the emergence of these sophisticated forms of experience. Such theoretical pluralism aligns with the pragmatist tradition’s resistance to reductionism but also incorporates insights from contemporary neuroscience about the mechanisms that enable experiential integration.

In summary, our hybrid neurophilosophical framework builds on Dewey’s foundational insights while incorporating contemporary perspectives from predictive processing, enactivism, and network neuroscience (Gallagher, 2017). This integration, which faces its own challenges that will have to be carefully tackled in future research, provides a potentially richer understanding of how supercomplex experiences emerge from the dynamic coordination of multiple systems operating across different scales of organization. By maintaining continuity with philosophical traditions while embracing neuroscientific evidence, we offer an account that aims at being both theoretically sophisticated and empirically grounded.

We acknowledge that the philosophical debate about the nature of experience is vast and extraordinarily complex, encompassing millennia of inquiry from ancient philosophy through phenomenology, philosophy of mind, and contemporary consciousness studies. Fully situating the notion of supercomplex experiences within this broader philosophical landscape would be a fascinating endeavor, requiring engagement with questions about qualia, the hard problem of consciousness, the relationship between first-person and third-person perspectives, and fundamental issues in ontology and epistemology, among many others. However, such a comprehensive philosophical treatment lies outside the scope of this paper, as it would constitute an entire study in its own right. For this reason, we have deliberately focused on the relatively narrow slice of this debate that most directly engages with the neural underpinnings of experience—specifically those philosophical frameworks that offer productive dialog with contemporary neuroscience. This focused approach allows us to develop a framework that is philosophically informed to some extent while remaining grounded in empirical evidence about how the brain supports these remarkable forms of human experience.

## Defining supercomplex experiences

Building on the illustrative examples presented above, we can now formulate a specific definition of supercomplex experiences:

Experiences that simultaneously engages multiple, hierarchically organized neural networks in a coordinated manner, requiring both: (1) the dynamic integration of multiple processing streams (sensory, emotional, cognitive, and social), and (2) the development of specialized neural architectures and conceptual frameworks for processing and communicating these integrated perceptions. Such experiences are characterized by their resistance to decomposition into simpler component processes while maintaining coherent gestalt properties that emerge from the interaction of their constituent elements.

The term “supercomplex” is introduced to distinguish these experiences from merely “complex” ones. While many everyday experiences involve multiple processes, what sets supercomplex experiences apart is their integrative nature and resistance to decomposition, combined with the need for specialized neural and conceptual architectures. The prefix “super-” denotes both the hierarchical organization (processing streams operating above and across multiple levels simultaneously) and the qualitative emergent properties that transcend mere complexity. Moreover, while simultaneous engagement of multiple processing streams is a feature of cognition more broadly meant (Colombetti, 2014; Pessoa, 2022), what distinguishes supercomplex experiences is not merely the presence of this simultaneous engagement but rather its degree, organization, and the specific demands it places on neural architecture.

This definition extends beyond existing conceptualizations in several important ways. Whereas theories of embodied cognition discuss multimodal integration (Barsalou, 2008), they typically don’t address the specialized neural and conceptual architectures required for domains like wine tasting or artistic performance. Unlike Damasio’s (1999) concept of “extended consciousness” or Edelman’s (2004) “higher-order consciousness,” which focus primarily on the temporal integration of experience, our concept of supercomplexity emphasizes the simultaneous engagement and coordination of multiple processing streams.

The concept also differs from existing frameworks in cognitive neuroscience that address multisensory integration (Stevenson et al., 2014) or cross-modal processing (Calvert, 2001; Driver and Noesselt, 2008). These frameworks explain how different sensory inputs are combined, but they do not fully capture the development of expertise-specific neural architectures that characterize supercomplex experiences. Similarly, while theories of expertise (Ericsson and Pool, 2016) discuss the development of specialized cognitive skills, they typically focus on domain-specific abilities rather than the integrated processing demands of supercomplex experiences.

As already remarked, our definition presents interesting links with Vernazzani’s (2023) recent account of “aesthetic looking,” which emphasizes how attention to aesthetic, expressive, or design properties shapes perception. However, whereas Vernazzani focuses primarily on how artworks modify subsequent perception, our account of supercomplex experiences addresses the broader question of how integrated experiences emerge through the coordination of multiple neural systems in real-time.

To decompose our definition in its basic constituent parts allows us to appreciate better how it fills an important gap in the literature. In particular, supercomplexity provides a conceptual framework for the understanding of experiences that simultaneously present a combination of characteristics, namely:

(1) Require simultaneous engagement of multiple neural networks in ways that cannot be reduced to sequential processing;
(2) demand the development of specialized neural architectures through extensive training and exposure;
(3) necessitate the creation of novel conceptual frameworks and specialized vocabularies for their communication;
(4) exhibit emergent properties that arise from the dynamic interaction of multiple processing streams;
(5) maintain coherent gestalt properties despite their internal complexity.

Importantly, we are not suggesting that these characteristics exist in binary form (present or absent), but rather that supercomplex experiences represent domains where all these dimensions are simultaneously present to a high degree. The wine tasting example illustrates how expertise in an experientially rich domain simultaneously transforms perception, conceptualization, and communication in ways that cannot be fully understood through any single existing theoretical framework.

A potential concern with our definition is that it might appear circular: we define supercomplex experiences in terms of specialized vocabularies and neural architectures and then use the presence of these features to identify supercomplex experiences. However, this concern misunderstands the explanatory relationship we are proposing.

The five criteria above are not merely descriptive features but point to specific causal mechanisms that explain why these phenomena co-occur in certain domains. The mechanism of predictive processing across multiple levels, combined with expertise-dependent network reorganization, provides a potential causal explanation for why domains characterized by supercomplex experiences develop such specialized vocabularies and neural architectures. This explanatory relationship moves beyond mere description to identify common generative mechanisms across seemingly disparate domains.

For example, consider the development of expertise in wine tasting. The specialized vocabulary that wine critics develop is not arbitrary but reflects the specific demands of communicating subtle perceptual discriminations that most people lack the conceptual framework to articulate. Similarly, the neural reorganization observed in wine experts is not separate from their perceptual capacities but enables the sophisticated integration of olfactory, gustatory, somatosensory, and conceptual processing that characterizes expert wine tasting.

This conceptualization therefore helps explain why certain domains, such as wine tasting, musical performance, and artistic creation, among others, require such extensive training and develop such specialized vocabularies. It also provides a theoretical foundation for understanding why these experiences cannot be adequately captured through existing frameworks of perception, expertise, or consciousness.

Furthermore, the concept of supercomplex experiences bridges important theoretical gaps between predictive coding approaches (Huang and Rao, 2011), theories of embodied cognition (Foglia and Wilson, 2013), and research on expertise development (Elvira et al., 2017). By emphasizing both the neural architectures required for processing these experiences and their resistance to decomposition, our framework contributes to an explanation of why certain domains require the development of such distinctive patterns of neural organization.

This definition also has important implications for understanding individual differences in the capacity to process and communicate supercomplex experiences. It suggests that expertise in domains characterized by such experiences involves not just the refinement of individual skills but the development of integrated processing capabilities that allow experts to navigate multiple streams of information simultaneously while maintaining coherent experiential wholes (Christensen et al., 2016; Boshuizen et al., 2020). However, it is important to stress that the development of specialized vocabularies reflects the communicative needs that arise from these experiences, not necessarily their subjective quality or depth. For instance, as Elkins et al. (2004) notes in discussing art viewing, specialized knowledge can sometimes “dull” direct emotional encounters with art by pointing attention to analytical rather than experiential aspects. This highlights an important tension within supercomplex experiences between analytical discrimination and phenomenological immersion, a tension that experts learn to navigate through their training and their personal journey (Tan et al., 2017).

We acknowledge that introducing a new term requires justification, but we believe that the notion of “supercomplex experiences” addresses a significant gap in the current cognitive neuroscience literature which has tended to study these phenomena through separate sub-disciplinary lenses. Domains like wine tasting, musical performance, and artistic creation have been studied extensively, but the shared characteristics that make these activities cognitively distinctive have not been adequately theorized. With this framework, we aim to enable a cross-fertilization between research programs that have traditionally remained separate, while providing a neuroscientifically grounded account of these integrated experiences.

## Boundary cases: distinguishing supercomplex from other types of experiences

To better delineate the boundaries of supercomplex experiences, it is instructive to examine cases that might appear complex but fail to meet one or more of our defining criteria. Such analysis helps clarify why supercomplexity requires the simultaneous satisfaction of all five conditions.

Consider first the experience of riding a bicycle. While this activity engages multiple sensory and motor systems simultaneously, requiring balance, spatial awareness, and coordination, it fails to meet our third criterion - the need for specialized vocabularies and conceptual frameworks. Once mastered, cycling can be effectively described using ordinary language and concepts (Ramachandran and Hubbard, 2001). Despite its procedural complexity, it does not generate the kind of specialized lexicons we observe in wine tasting or musical performance. This illustrates how simultaneous engagement of multiple neural networks alone is insufficient for supercomplexity.

Amateur playing of a video game provides another counterexample. While modern games can create deep-layered multisensory experiences engaging attention, emotion, and motor control, they are purposefully designed to be user-friendly enough to not need the meeting of our second criterion – the requirement for specialized neural architectures developed through extensive training. Casual gamers can quickly achieve the competence they need to enjoy playing the game, without developing the kind of expertise-specific neural reorganization observed in professional musicians or sommeliers, or in professional gamers. The experience, while engaging, remains within the bounds of ordinary cognitive processing capabilities.

The experience of chronic pain presents another interesting contrarian case. Chronic pain involves complex interactions between sensory, emotional, and cognitive systems, and can lead to the development of specialized neural architectures (Barroso et al., 2021). However, it fails to meet our fourth criterion: the emergence of coherent gestalt properties from dynamic interactions. Instead, chronic pain often represents a dysregulation of normal processing rather than the emergence of higher-order experiential properties (Ferraro et al., 2022).

Consider also the case of routine social interactions (Redcay, 2008; Redcay and Schilbach, 2019), such as a typical business meeting. While these situations involve multiple participants and social signals, they fail to meet our first criterion: the requirement for truly simultaneous engagement of multiple neural networks in ways that resist decomposition. Such interactions can typically be broken down into sequential components (turn-taking in conversation, reading facial expressions, processing verbal content) without losing their essential character. This contrasts with the irreducible simultaneity required, say, in orchestral conducting or improvisational dance.

Even expertise in highly technical fields may not qualify as supercomplex if it lacks certain critical elements. Consider a skilled computer programmer. While programming requires extensive training and can involve complex problem-solving, it typically engages cognitive systems sequentially rather than simultaneously and does not require the kind of dynamic, real-time integration characteristic of supercomplex experiences. The programmer can break down problems into discrete steps and tackle them sequentially, unlike, say, a chef who must simultaneously monitor and respond to multiple food preparation lines while maintaining the overall control of the execution.

These examples illustrate how supercomplexity emerges only when all five criteria are simultaneously satisfied. The absence of any single criterion results in experiences that, while potentially complex or challenging, lack the distinctive characteristics that define truly supercomplex experiences. This understanding helps explain why domains characterized by supercomplex experiences develop such distinctive patterns of expertise and require such extensive training: they demand the simultaneous satisfaction of multiple conditions that rarely co-occur in ordinary experience.

It’s worth emphasizing that supercomplexity exists on a continuum rather than as a binary characteristic. The examples above illustrate cases that clearly fall short on particular dimensions, but there are many activities that fall into gray areas. Professional sports, for instance, involve many characteristics of supercomplex experiences but may vary in the degree to which they develop specialized conceptual frameworks. Similarly, medical diagnosis shares many features with supercomplex experiences but varies across specialties in the degree of simultaneous versus sequential processing required. Our framework provides a theoretical basis for examining these variations systematically rather than merely classifying experiences as supercomplex or not.

## Key domains of supercomplex experiences

Having established the defining criteria for supercomplex experiences, we can now examine exemplary domains where these experiences manifest most clearly. Rather than attempting a comprehensive taxonomy at this early stage of theoretical development, we will focus on identifying key domains where the five criteria of supercomplexity are particularly evident. This approach allows us to highlight the shared characteristics across diverse activities while acknowledging the need for further empirical validation.

*Artistic performance* represents a primary domain of supercomplex experiences, characterized by the real-time integration of technical execution, emotional expression, and social awareness (Ramírez-Moreno et al., 2023). Jazz improvisation exemplifies this domain, requiring musicians to simultaneously monitor multiple instrumental lines while maintaining overall interpretive coherence and responding to fellow performers (Berliner, 1994; D’Ausilio et al., 2015). The neural demands of such performances engage auditory processing, motor control, emotional systems, and social cognition in ways that cannot be reduced to sequential processing, creating what Schön (2017) described as thinking in action, a form of profession-specific embodied cognition that transcends explicit rule-following.

*Sensory evaluation* constitutes another key domain, particularly evident in activities like wine tasting, perfumery, and expert food assessment. These practices involve the simultaneous engagement of multiple sensory processing streams (olfactory, gustatory, tactile) while integrating conceptual knowledge and memory (Honoré-Chedozeau et al., 2024). The vocabulary developed by experts in these fields, and exemplified in the wine reviews presented earlier, suggests how these domains necessitate specialized conceptual frameworks that go beyond ordinary language, enabling the communication of subtle distinctions that would otherwise remain ineffable.

*Artistic creation* represents a third significant domain, encompassing activities like painting, sculpture, and musical composition. These creative processes involve continuous feedback between perception, action, and evaluation, with the artist simultaneously attending to technical execution, compositional structure, and expressive qualities (Zeki, 2001). Neuroimaging studies reveal that artistic creation engages both focused attention networks and the default mode network in distinctive patterns of interaction that differ from ordinary cognitive tasks (Beaty et al., 2018).

*Ritualistic and ceremonial leadership* constitutes a fourth domain where supercomplexity is evident. Leading religious rituals, traditional ceremonies, or formal cultural practices requires simultaneous attention to procedural details, symbolic meanings, emotional states of participants, and temporal pacing (Xygalatas, 2022). These activities exemplify how supercomplex experiences often have significant social dimensions, creating shared experiential spaces that integrate individual cognitive processes into collective meaning-making (Xygalatas et al., 2024).

Understanding these domains helps illuminate why certain activities develop distinctive expertise patterns and specialized training methods. The examples highlighted above all involve the simultaneous satisfaction of our five criteria for supercomplexity, demonstrating how these activities require integrated processing across multiple neural networks while maintaining coherent experiential wholes. By focusing on these exemplary domains rather than attempting a comprehensive taxonomy, we aim to provide clear reference points for further theoretical development and empirical research.

We can summarize the previous discussion with the following Table 1, which shows how the various experience criteria that define supercomplexity apply to the examples above:

**TABLE 1:** Application of supercomplex experience criteria across domains.

Criterion	Wine tasting	Musical performance	Painting	Religious ritual
1. Multiple neural networks engaged simultaneously	Integration of gustatory, olfactory, somatosensory, and memory systems with conceptual knowledge	Coordination of auditory, motor, emotional, and social processing with temporal prediction	Integration of visual, motor, memory, and emotional systems with conceptual knowledge	Coordination of sensory, emotional, social, and symbolic processing systems
2. Specialized neural architectures through training	Enhanced representational specificity in sensory regions; strengthened connections between sensory and semantic networks	Structural and functional changes in auditory-motor systems; enhanced predictive modeling	Modifications to visual-motor coordination systems; integration of default mode and executive networks	Heightened interoceptive awareness; enhanced coordination between attention and emotional systems
3. Specialized vocabularies and conceptual frameworks	Elaborate lexicon of sensory descriptors and metaphors; structured evaluation frameworks	Technical vocabulary for musical elements; interpretive frameworks for expression	Visual design principles; conceptual frameworks for compositional relationships	Symbolic vocabulary; ritualistic frameworks with established meanings
4. Emergent properties from dynamic interactions	Flavor profiles emerge from interactions between discrete chemical compounds and contextual factors	Musical meaning emerges from interactions between notes, rhythms, and dynamic variations	Visual coherence emerges from interactions between individual elements and compositional relationships	Collective meaning emerges from interactions between individual actions and shared symbolic frameworks
5. Coherent gestalt properties	Unified taste experience that transcends individual flavor components	Unified musical interpretation that transcends individual technical elements	Unified visual composition that transcends individual brushstrokes	Unified ritual experience that transcends individual symbolic actions

An important distinction emerges between two broad categories of supercomplex experiences: productive/creative experiences (such as musical performance, painting, and ritual leadership) and receptive/analytical experiences (such as wine tasting, art appreciation, and certain forms of listening). While both categories satisfy our five criteria for supercomplexity, they engage neural networks in different patterns. Productive experiences typically involve stronger activation of motor planning and execution systems alongside creative generation networks, while receptive experiences emphasize discriminative perception and evaluative judgment. This distinction helps address the concern that wine tasters engage in “secondhand communication” while performers communicate with the audience through the same artistic act. Both involve specialized neural architectures and conceptual frameworks but deploy them differently. Wine tasters translate perceptual experiences into language, whereas performers translate conceptual intentions into aesthetic actions (Frascaroli et al., 2024). Despite these differences, both types of experience resist decomposition into simpler processes and require a sophisticated integration across multiple neural systems.

This comparative analysis therefore reveals both commonalities and differences across supercomplex domains. While all four examples satisfy each criterion, they do so in different ways. Wine tasting primarily involves analytical-receptive processes, musical performance and painting recruit productive-creative processes. Religious ritual uniquely emphasizes collective coordination. These differences highlight the diversity within the broader category of supercomplex experiences while confirming the usefulness of these five criteria for identifying them.

## A unified theoretical framework: embodiment, interoception, and supercomplex experience

The exploration of supercomplex experiences requires a theoretical framework that bridges multiple levels of analysis, from neural mechanisms to embodied action to cultural practices. This section integrates insights from embodiment theory, interoceptive neuroscience, and network dynamics to provide a comprehensive account of how these sophisticated experiences emerge and operate.

Modern neuroscience has revealed mechanisms that help explain how the brain integrates information across distributed networks, building on early insights from Gestalt psychology about holistic organization (Koffka, 1935). While Gestalt psychologists could only speculate about underlying mechanisms, contemporary network neuroscience demonstrates how the brain achieves the integration of multiple processing streams through dynamic coordination of large-scale networks (Shine et al., 2019). This integration occurs not in isolation but through the fundamental embeddedness of neural processes in body-environment interactions (Gallagher, 2020).

Central to this framework is the role of interoception (the neural processing of internal bodily signals) in laying the embodied foundation for supercomplex experiences. The interoceptive system, centered in the anterior insula and anterior cingulate cortex, operates in parallel with but distinct from the exteroceptive system that processes external sensory information. Craig’s (2003) pioneering research revealed that primates possess a distinct cortical representation of homeostatic afferent activity in the insula, reflecting the physiological condition of all body tissues. This system provides continuous information about the body’s internal state, from heart rate and respiration to hunger and arousal, creating a sort of “sentient self” that grounds all conscious experience.

The integration of interoceptive and exteroceptive streams occurs through the salience network, which determines the behavioral relevance of both internal and external signals. This integration is not merely additive but generates emergent properties that characterize embodied experience (Merleau-Ponty, 1962). When a wine expert evaluates a wine, for instance, the experience emerges from the integration of gustatory and olfactory sensations with subtle interoceptive signals about arousal, pleasure, and bodily comfort. The anterior insula serves as a crucial hub for this integration, showing enhanced activation and connectivity in experts who have learned to attend to and interpret these subtle bodily signals.

Understanding supercomplex experiences also requires considering how embodiment and action fundamentally shape neural processing. Multiple theoretical frameworks converge on the insight that perception and cognition cannot be separated from action. Neuroscientific evidence demonstrates that neural processing is profoundly influenced by action-perception cycles (Madl et al., 2011; Musall et al., 2019). Studies of sensorimotor contingencies show that perception involves not just passive reception, but active exploration guided by predictions about how sensory inputs will change with movement (O’Regan and Noë, 2001).

This action-oriented perspective illuminates why supercomplex experiences often involve sophisticated forms of embodied engagement. The musician’s experience emerges not just from auditory processing but from the intricate coordination of breathing, posture, and fine motor control. The painter’s perception of color and form is inseparable from the physical act of applying the paint to the canvas. These are not merely motor accompaniments to cognitive processes but fundamental constituents of the experience itself.

The quality of experience depends critically on what has been termed “skilled intentionality”, i.e., the capacity to be selectively attuned to relevant affordances in one’s environment (Bruineberg et al., 2018). This attunement develops through extensive practice that refines both perceptual sensitivity and motor responsiveness. The fronto-parietal attention network implements this selective processing by modulating activity in sensory cortices based on behavioral goals and learned relevance. In experts, this modulation becomes increasingly sophisticated, allowing rapid, flexible attention to subtle features that novices simply cannot detect.

The neuroscientific concept of allostasis provides another crucial component of our framework. Rather than maintaining fixed physiological setpoints, the brain engages in anticipatory regulation, continuously adjusting bodily systems based on predicted future demands (Sterling, 2012). The anterior insula, anterior cingulate cortex, and ventromedial prefrontal cortex coordinate this predictive regulation, integrating interoceptive signals with environmental cues and memory to prepare the body for upcoming challenges. In supercomplex experiences, this allostatic regulation becomes particularly sophisticated, as experts learn to modulate their physiological states to optimize performance.

The hierarchical organization of predictive processing provides a mechanism for integrating these multiple components (see Appendix for a methodological note on predictive processing). The brain maintains generative models at multiple levels, from basic sensory predictions to abstract conceptual frameworks. In supercomplex experiences, these levels become tightly coordinated, with predictions at each level constraining and being constrained by the others. The wine expert’s brain simultaneously predicts basic gustatory sensations, flavor evolution over time, and how the current wine fits within broader categorical frameworks. Prediction errors at any level can trigger updates throughout the hierarchy, creating the dynamic responsiveness that characterizes expert performance.

Damasio’s convergence-divergence hypothesis offers insight into how this integration creates unified experiences from diverse inputs. Association cortices bind information from lower sensory regions with visceral, motor, and emotional inputs through zones of convergence that operate at multiple hierarchical levels (Man et al., 2013). This creates what Damasio calls “images”: integrated representations that include not just sensory features but their emotional and bodily significance. In supercomplex experiences, these images achieve remarkable richness and dimensionality through the enhanced connectivity between convergence zones that develops with expertise.

The dynamic coupling between perception and action in supercomplex experiences extends beyond individual body-brain systems to include tools, environments, and other people. Studies of tool use reveal how the brain’s body schema dynamically expands to incorporate instruments, with the posterior parietal cortex showing plasticity in representing the extended body (Maravita and Iriki, 2004). For the violinist, the instrument becomes a genuine extension of their body schema, incorporated into the predictive models that guide performance. Similarly, the painter’s brush, the chef’s knife, or the sommelier’s glass become integrated into embodied networks of perception and action.

This extended embodiment has profound implications for understanding expertise. Masters in any domain develop not just internal neural changes but transformed relationships with their tools and environments. The wine expert’s interaction with the glass (the swirling, sniffing, and tasting rituals) represents learned patterns of sensorimotor engagement that reliably evoke particular perceptual states. These patterns become so deeply embodied that they operate below conscious awareness, yet they fundamentally shape the resulting experience.

The social dimensions of embodiment add another layer to our framework. Mirror neuron systems and other mechanisms of sensorimotor simulation allow us to partially share others’ embodied states (Gallese, 2005, 2014; Gallese and Sinigaglia, 2011). In collective supercomplex experiences, this creates possibilities for genuine inter-bodily resonance. Musicians in ensemble performance show synchronized breathing, heart rate variability, and movement patterns that go beyond mere coordination to create shared physiological states. This biological synchrony may provide the foundation for the sense of unity and transcendence often reported in peak collective experiences.

Cultural practices shape embodied experience in profound ways, creating “cultural body schemas” that influence perception and action. Different wine-tasting traditions, for instance, emphasize different patterns of attention and different vocabularies for describing experience. These cultural variations become literally embodied through neural plasticity, creating population-level differences in perceptual capabilities and expressive frameworks, e.g., the superior average coffee-tasting skills of people from “coffee societies” where drinking coffee is a daily, repeated ritual. Understanding supercomplex experiences thus requires attention not just to universal neural mechanisms but to how these mechanisms are shaped by cultural learning.

The aesthetic dimension of supercomplex experiences, long considered separate from their cognitive aspects, emerges naturally from this embodied framework. Aesthetic feelings arise from the dynamic interplay between sensory processing, bodily states, and predictive models. The sense of rightness when a musical phrase resolves, the pleasure of balanced composition in visual art, or the satisfaction of harmonious flavors all reflect successful prediction and fluid action-perception coupling. Rather than being mere subjective additions to cognitive processing, these aesthetic feelings serve as crucial guides for navigation through complex experiential spaces.

This unified framework presents supercomplex experiences as paradigmatic examples of human cognition operating at its most integrated and sophisticated level. They demonstrate how the brain’s predictive architecture, the body’s sensorimotor capacities, and the environment’s affordances can become so tightly coupled that they operate as a single cognitive system. The expertise that enables these experiences represents not just neural changes but a fundamental reorganization of the person-environment relationship, creating new possibilities for perception, action, and meaning-making.

Understanding these mechanisms has implications extending far beyond the specific domains we have examined. If supercomplex experiences represent human cognition operating in its most integrated mode, then studying them may reveal principles relevant to cognition more broadly. The frameworks developed for understanding wine tasting or musical performance might inform our conceptualization of how integration occurs in other domains, from scientific discovery to social interaction to contemplative practice. By taking supercomplex experiences seriously as objects of scientific study, we might open new windows into the nature of human consciousness and capability.

## The integration of aesthetics and cognition in supercomplex experiences

In supercomplex experiences, aesthetic and cognitive processing are tightly integrated through shared neural architectures. Even seemingly pure aesthetic judgments engage sophisticated cognitive processing streams, while apparently pure cognitive tasks involve aesthetic components of coherence and form (Pelowski et al., 2017). This integration is particularly evident in the dynamic interaction between the default mode network and executive control systems during complex tasks requiring both creative generation and analytical evaluation. The development of expertise in domains characterized by supercomplex experiences provides crucial insight into how aesthetic and cognitive processing become increasingly entrained through experience, creating unified processing streams that handle both immediate experiential qualities and abstract analytical understanding.

This integration has important implications for understanding the nature of both aesthetic and cognitive processing. The anterior insula and anterior cingulate cortex appear crucial for integrating emotional and cognitive aspects of experience, suggesting that this integration is a basic feature of how the brain processes complex information rather than a special case. The role of prediction in both aesthetic and cognitive processing provides another crucial link, as aesthetic pleasure may arise from the successful resolution of prediction errors at multiple levels, whereas cognitive understanding involves the construction and refinement of predictive models.

The integrative function of supercomplex experiences offers useful clues as to why certain learning experiences are more effective than others. Educational approaches that engage both aesthetic and cognitive faculties may better mirror how the brain naturally processes complex information, suggesting new approaches to pedagogy that deliberately engage multiple processing streams rather than attempting to separate “creative” from “analytical” learning. This integration appears to be implemented through the coordinated activity of large-scale brain networks, particularly through the interaction of the salience network with both task-positive and task-negative networks.

Understanding how supercomplex experiences integrate aesthetic and cognitive dimensions also helps explain why domains characterized by such experiences often develop distinctive cultural practices and training methods. These methods typically cultivate both immediate perceptual sensitivity and sophisticated conceptual frameworks, recognizing implicitly that expertise requires sophisticated toggling between cognitive and aesthetic modes. The neural plasticity observed in experts suggests that the brain can develop specialized architectures supporting this dynamic integration through sustained engagement with experiences that demand both aesthetic sensitivity and cognitive sophistication at high levels.

## Network dynamics in supercomplex experiences

As already remarked, the neural basis of supercomplex experiences emerges from the dynamic interaction of three core brain networks, each with distinct but complementary roles in processing complex information. These networks (the salience network, the default mode network, and the central executive network) form the fundamental architecture supporting sophisticated cognitive integration (Goulden et al., 2014; Sullivan et al., 2018). Understanding their individual functions and coordinated interactions provides crucial insight into how the brain manages the simultaneous processing demands that characterize supercomplex experiences.

The salience network, anchored in the anterior insula and anterior cingulate cortex, serves as the brain’s dynamic gateway for managing the flow of information between internal and external processing modes (Steimke et al., 2017; Snyder et al., 2021; Pereira et al., 2024). Through extensive connections with both sensory regions and higher-order cognitive networks, it identifies behaviorally relevant stimuli and triggers appropriate network-level reconfigurations. This network’s unique position allows it to integrate interoceptive signals with external sensory information, determining which inputs deserve attentional resources and how they should be processed (Uddin, 2015).

In the context of supercomplex experiences, the salience network plays a particularly crucial role. During musical performance, for instance, it enables rapid switching between different aspects of experience based on their momentary relevance, from technical execution to expressive nuance to ensemble coordination. The anterior insula, a key hub within this network, shows enhanced activation in experts compared to novices, reflecting its role in integrating the multiple streams of information that must be coordinated during complex performance (Wieck et al., 2010; Chong et al., 2017; Ueno et al., 2020).

The default mode network, traditionally associated with self-referential processing and mind-wandering, features specialized functions in the context of supercomplex experiences. Centered on the medial prefrontal cortex, posterior cingulate cortex, and angular gyrus, this network typically shows decreased activation during externally focused tasks. However, recent research has revealed a more nuanced picture of its role in complex cognition (Vatansever et al., 2017; Grieder et al., 2018). Rather than simply “deactivating” during demanding tasks, the default mode network actively contributes to the integration of personal knowledge, semantic memory, and ongoing experience.

Smith et al. (2018) demonstrated that the default mode network shows enhanced activity during cognitive transitions, particularly during task switches and restarts after brief rest periods. This suggests its involvement not just in internally directed cognition but in the active reconfiguration of cognitive context during transitions between different task demands. Menon’s (2023) comprehensive review further elaborates how the default mode network integrates multiple cognitive operations to construct internal narratives, with its hub properties facilitating both external and internal event-driven network switching.

Research on aesthetic experiences has particularly highlighted the default mode network’s sophisticated role. Vessel et al. (2012, 2013, 2019) found that aesthetic appreciation engages this network in ways that differ fundamentally from ordinary object processing. When viewing highly pleasing artworks, default mode network activity shows a characteristic pattern of engagement that tracks the viewer’s internal state rather than merely responding to stimulus properties. Belfi et al. (2019) extended these findings by showing that for aesthetically appealing artworks, default mode network activity returns to baseline in a manner time-locked to image offset, while timing remains inconsistent for non-pleasing art. These findings suggest that the network dynamically tracks the viewer’s engagement, integrating perceptual, emotional, and conceptual aspects of aesthetic appreciation.

The central executive network, anchored in the dorsolateral prefrontal cortex and posterior parietal cortex, implements cognitive control and attention allocation (Cieslik et al., 2013; Daigle et al., 2022). This network modulates activity in other brain regions based on current goals and task demands, enabling the flexible deployment of cognitive resources. In supercomplex experiences, it coordinates the integration of multiple processing streams while maintaining focus on relevant aspects of experience. The network’s role extends beyond simple task execution to include the sophisticated understanding of complex experiences (Errante and Fogassi, 2019).

The interaction between these networks exhibits a sophisticated temporal dynamics characterized by metastable patterns of coordination. Rather than maintaining fixed connectivity patterns, these networks show fluid transitions between different configurations, allowing for both integrated processing and specialized computation as needed (Tognoli and Kelso, 2014; Capouskova et al., 2022). This metastability, a dynamic regime that maintains a balance between integration and segregation, proves crucial for supercomplex experiences. It enables a simultaneous engagement of multiple processing modes while maintaining overall coherence.

Expert performers demonstrate an enhanced metastability in networks relevant to their domain of expertise. This enhanced flexibility allows more sophisticated integration of multiple processing streams compared to novices (Bruineberg et al., 2021). The development of this network flexibility appears to be a key marker of expertise acquisition, reflecting the brain’s ability to adaptively reconfigure its functional architecture based on task demands.

Hub regions play a critical role in orchestrating these network interactions. The anterior insula serves as a particularly important node, facilitating communication between networks through its unique anatomical position and connectivity pattern (Menon and Uddin, 2010). It enables rapid reconfigurations of network relationships based on current processing demands, acting as a switch that can redirect information flows between different functional systems. Other crucial hubs include the posterior cingulate cortex, which helps integrate information across networks (Pearson et al., 2011), and the anterior cingulate cortex, which coordinates between control and attention systems (Weissman et al., 2003; Bryden et al., 2011).

These network dynamics operate across multiple temporal scales, adding another layer of complexity to their coordination. Fast-scale interactions, occurring over milliseconds to seconds, support immediate integration and response generation—crucial for real-time performance in domains like music or sports. Slower-scale changes in connectivity patterns, unfolding over minutes to hours, underlie the development and maintenance of expertise. Even longer timescales, spanning weeks to years, reflect the structural changes that accompany expertise development (Bassett et al., 2015; Shine et al., 2016).

The maintenance of balanced integration and segregation among neural systems emerges as a critical feature of healthy network function. Too much integration can lead to undifferentiated processing, where distinct types of information become conflated. Conversely, too much segregation can prevent effective coordination between specialized systems. Supercomplex experiences appear to require sophisticated metastable dynamics that calibrate and maintain this crucial balance, allowing for both specialized processing in domain-specific networks and integrated processing across systems (Cross et al., 2021; Jang et al., 2024).

Individual differences in network organization contribute significantly to variations in how people process and master supercomplex experiences. Some individuals show greater natural network flexibility or more optimal hub organization, potentially predisposing them to excel in particular domains (Tompson et al., 2018). These individual differences interact with training and experience to shape the development of expert performance capabilities. Research suggests that while baseline network organization may influence initial aptitude, the plasticity of these networks implies that sustained training can overcome many initial limitations (Cole et al., 2013; Pinho et al., 2014; Amoruso et al., 2017).

The adaptability of network dynamics proves crucial for both learning and ongoing skill refinement. As individuals encounter new challenges within their domain of expertise, their network architecture can reconfigure to accommodate novel processing demands and maintain established capabilities at the same time. This plasticity enables the progressive refinement of expertise while preserving the ability to handle unexpected situations: a hallmark of true mastery in complex domains (Caley et al., 2014; Frie et al., 2019).

Network flexibility, defined as the ability to rapidly and efficiently reconfigure functional connections based on task demands, emerges as perhaps the most crucial characteristic for successful engagement with supercomplex experiences. Expert performers show particularly enhanced network flexibility in regions that serve as specialized connector hubs between different functional networks (Bertolero et al., 2015; Jeon and Friederici, 2017; Binder et al., 2017). Studies of musicians have demonstrated how network flexibility in temporal and prefrontal regions predicts performance quality, suggesting its fundamental importance for expertise expression (Pallesen et al., 2010).

In collective settings, these network dynamics extend beyond individual brains to create patterns of inter-brain synchronization. During joint performance or coordinated action, participants show synchronized activity in regions associated with social cognition and joint attention, with the strength of this synchronization predicting successful coordination (Dumas et al., 2010; Valencia and Froese, 2020; Shiraishi and Shimada, 2021; Zhou et al., 2022). This suggests that the network architecture supporting supercomplex experiences can extend to include multiple brains operating in coordinated ways, creating emergent properties at the group level that transcend individual capabilities.

This sophisticated network architecture ultimately enables the key characteristics of supercomplex experiences: simultaneous engagement of multiple processing streams, resistance to decomposition into simpler components, emergence of coherent gestalt properties, and development of specialized frameworks for understanding and communication. The dynamic interaction between these networks, their ability to rapidly reconfigure based on task demands, and their capacity for both specialized and integrated processing create the neural foundation for experiences that transcend simple categorization and require sophisticated expertise for their full appreciation. Understanding these network dynamics not only yields insight on the neural basis of expertise but also suggests possible principles for optimizing training and performance in complex domains.

## Neural implementation of supercomplex experiences

To understand how supercomplex experiences emerge in real-time, we must examine the cascading neural processes that transform raw sensory inputs into richly structured experiences. This transformation involves multiple levels of processing that operate simultaneously rather than sequentially, creating the integrated phenomenology that characterizes these experiences. Here we trace this implementation through its key stages, focusing on how expertise shapes each level of processing.

The initial stage involves multilevel sensory processing and integration. When sensory information first reaches specialized receptors, it triggers parallel processing streams that will ultimately converge to create unified percepts. In wine tasting, for instance, chemoreceptors in the tongue and retro-nasal cavity transduce chemical compounds into neural signals transmitted to primary gustatory and olfactory cortices, while somatosensory systems simultaneously encode texture, temperature, and mouthfeel. These diverse signals converge in the anterior insula and orbitofrontal cortex, the critical hubs for multisensory integration (Small and Prescott, 2005).

The anterior insula plays a particularly pivotal role by integrating interoceptive signals with exteroceptive sensory information. Neuroimaging studies reveal that expert wine tasters show enhanced functional connectivity between the anterior insula and primary sensory regions compared to novices, suggesting more efficient integration of multimodal sensory information (Castriota-Scanderbeg et al., 2005; Pazart et al., 2014). This enhanced connectivity reflects the specialized neural architecture that develops through training, enabling experts to construct more fine grained and integrated sensory experiences.

Simultaneous with sensory integration, the anterior temporal lobe, a critical hub for semantic integration, matches emerging sensory patterns to stored knowledge. Pazart et al. (2014) found that sommeliers show greater activation in the left anterior temporal lobe compared to novices when evaluating wines, reflecting their ability to rapidly categorize sensory patterns based on extensive domain knowledge. The hippocampus and associated medial temporal regions contribute contextual information, activating relevant autobiographical and episodic memories that enrich the perceptual experience.

As the experience unfolds, predictive processing mechanisms continuously update neural representations through hierarchical generative models. These models, centered in the orbitofrontal cortex and anterior insula, yield predictions about expected sensory inputs and update them based on incoming sensory evidence. The precision-weighting of prediction errors, determining which discrepancies between predictions and sensory inputs drive model updating, is adaptively modulated by expertise-dependent expectations. For expert wine tasters, this predictive processing operates across multiple timescales simultaneously: tracking immediate sensory profiles, monitoring flavor evolution over seconds or minutes, and contextualizing the current wine within broader knowledge frameworks.

A distinctive feature of supercomplex experiences is the generation of cross-modal metaphors to communicate sensory experiences that resist literal description. The angular gyrus supports the creation of these metaphors by mapping across sensory modalities, allowing experts to describe wines in terms of texture (”velvety tannins”), architectural features (”structured”), or emotional qualities (”brooding”). This metaphorical mapping engages language-related regions in the left inferior frontal gyrus and ventrolateral and posterior temporal cortex, which again show enhanced functional connectivity with sensory integration areas in experts compared to novices.

Perhaps most remarkably, these multiple processes (perception, evaluation, memory retrieval, language generation) occur simultaneously rather than sequentially. This simultaneity, implemented through the metastable dynamics described in our network dynamics section, represents a defining feature of supercomplex experiences. The prefrontal cortex orchestrates this integration, with different subregions contributing distinct functions: the orbitofrontal cortex encodes value and expected outcomes, the dorsolateral prefrontal cortex maintains working memory representations, and the anterior prefrontal cortex coordinates long-term goals with immediate perceptual and evaluative processes.

The development from novice to expert involves a fundamental reorganization of these implementation processes. Novices initially show greater activation in primary sensory regions and rely heavily on explicit analytical strategies. As expertise develops, processing becomes more efficient, with greater involvement of integrative regions and enhanced functional connectivity between sensory, semantic, and language areas. This shift reflects not just enhanced efficiency but a qualitative change in how information is processed: from sequential analysis to simultaneous integration.

Understanding this neural implementation helps us appreciate why supercomplex experiences require extensive training. The sophisticated coordination between neural systems does not emerge spontaneously but develops through a progressive reorganization in response to purposeful domain-specific training. The neural architectures supporting these experiences must be cultivated through extended engagement with appropriate exemplars, explaining why mastery in domains like wine tasting, musical performance, or artistic creation typically requires years of dedicated practice.

The resistance of supercomplex experiences to decomposition into simpler processes becomes clear when we consider this implementation. The emergent properties arise from the dynamic coordination of multiple systems, such that isolating individual components disrupts the very coordination that constitutes the experience. This resistance to decomposition is not merely conceptual but is grounded in the metastable dynamics of the neural systems that implement these experiences, where the whole truly becomes greater than the sum of its parts.

## Expertise development in supercomplex experiences: exemplification and neural plasticity

As already anticipated, the development of expertise in domains characterized by supercomplex experiences involves a profound reorganization of both perceptual capacities and neural architecture. This transformation occurs through the interplay of exemplification processes, whereby specific instances highlight and make salient particular properties, and experience-dependent neural plasticity that reshapes brain structure and function. Understanding this developmental trajectory requires an examination of both the pedagogical mechanisms that guide learning and the neurobiological changes that support increasingly sophisticated performance.

Following Vernazzani (2023), we propose that Goodman’s (1976, 1984) and Elgin’s (1996, 2017) concept of exemplification provides a powerful framework for understanding how individuals initially develop their capacity for supercomplex experiences. In Goodman’s formulation, exemplification involves “reference by displaying” with an “object-to-feature” direction. An exemplar both possesses certain properties and refers to those properties by highlighting them in particularly representative ways, such as a color chip in a paint store that points our attention to a specific shade of blue, making it possible to recognize it if found again elsewhere.

Vernazzani (2023, p. 430) provides a precise definition that helps us understand this process: “EXEMPLIFICATION: An item O (the exemplar) is a symbol that exemplifies F-ness in a context c where it plays an intended function f if: (a) O possesses (literally or metaphorically) F, and (b) O refers to F-ness, i.e.: (b.1) O embodies F in such a way as to highlight it, i.e., as to draw S’s informed attention to O’s being F; and, (b.2) O makes thereby S epistemically aware of F-ness.”

This definition helps us clarify how expertise develops in supercomplex domains. Expert wine tasters, for instance, are exposed to carefully selected exemplars during their training that highlight specific properties of wines. These exemplars both possess the properties in question and are presented in ways that draw attention to those properties, making the trainee epistemically aware of them. Similarly, musicians learn to recognize subtle expressive properties through exposure to exemplary performances that highlight specific interpretive choices, while visual artists develop perceptual expertise through studying works that exemplify compositional strategies, color relationships, or expressive techniques, and so on.

What makes exemplification particularly powerful as a training mechanism is that it provides what can be called “perceptual scaffolding” (Sterelny, 2010) for the development of expertise. The exemplar does not merely possess the relevant properties but embodies them in ways that guide attention, making those properties more salient and easier to detect. At the neural level, this scaffolding process shapes attentional networks in ways that facilitate the detection of domain-relevant properties. Artists commonly exploit basic visual phenomena that draw attention to particular regions of their works, scaffolding the viewer’s perceptual experience in ways that highlight specific properties. In the case of wine tasting, exemplary wines are often selected to demonstrate specific characteristics in isolation before more complex exemplars are introduced. This progressive scaffolding helps shape both the perceptual and conceptual frameworks that eventually enable supercomplex experiences.

The process through which exemplification leads to expertise involves three interrelated mechanisms operating at both cognitive and neural levels. First, perceptual tuning occurs as exposure to well-chosen exemplars gradually modifies perceptual systems to detect properties that were previously imperceptible or un-discriminable. This involves changes in the response properties of neurons in sensory processing regions, enhancing sensitivity to domain-relevant features. Longitudinal neuroimaging studies reveal that such tuning manifests as increased gray matter volume in relevant sensory areas and enhanced functional responses to domain-specific stimuli (Herholz and Zatorre, 2012).

Second, alongside perceptual tuning, exemplars help build conceptual frameworks that organize perceptual information. The specialized vocabularies that experts develop emerge from this process, providing conceptual structures that both reflect and shape perceptual discriminations. This conceptual development is supported by strengthened functional connectivity between sensory regions and areas involved in semantic processing, particularly the anterior temporal lobe and angular gyrus (Pazart et al., 2014).

Third, through repeated exposure to exemplars that highlight specific properties, attentional networks learn to prioritize domain-relevant information. This attentional training is evident in studies showing how experts’ eye movements differ from those of novices when viewing domain-specific stimuli (Vogt and Magnussen, 2007). Neural evidence reveals an enhanced flexibility in network reconfiguration, particularly in regions serving as connector hubs between different functional networks (Bassett and Mattar, 2017).

These three mechanisms work synergistically to transform what were initially simple perceptual encounters into increasingly structured, sophisticated supercomplex experiences. As perceptual systems become more sensitized to domain-relevant properties, conceptual frameworks more elaborate, and attentional patterns more refined, the subject gradually develops the capacity for integrated experiences that were previously inaccessible.

The neural implementation of this developmental process involves significant reorganization at multiple levels. At the level of sensory processing, expertise is associated with enhanced neural tuning and representational specificity in relevant sensory cortices. Professional musicians show increased gray matter volume in auditory regions, along with enhanced functional responses to musical stimuli. Similarly, expert wine tasters demonstrate more refined neural representations of wine-related odorants in olfactory processing regions. These changes reflect experience-dependent plasticity that enables more sophisticated discrimination of domain-relevant stimuli.

Beyond local sensory changes, expertise involves the development of specialized functional circuits that support integrated processing across multiple domains. Consider professional perfumery, where experts must simultaneously analyze, create, and communicate about extraordinarily complex olfactory experiences. Master perfumers show enhanced functional connectivity between primary olfactory cortex and higher-order integration regions, particularly in the orbitofrontal cortex and anterior insula (Delon-Martin et al., 2013). This enhanced connectivity reflects not just improved sensory discrimination but the ability to integrate olfactory information with conceptual knowledge, emotional associations, and linguistic categories.

The development of expertise also modifies how the brain implements predictive processing. Through extensive experience, experts develop increasingly sophisticated hierarchical models that capture subtle statistical regularities in their domain. These enhanced predictive models are implemented through strengthened top-down connections from prefrontal and parietal regions to sensory cortices (Cheung and Bar, 2012). Expert radiologists, for instance, show more efficient activation patterns in visual processing regions when viewing medical images, along with enhanced connectivity between visual areas and regions involved in decision-making (Harley et al., 2009).

Critically, expertise development in supercomplex domains shows a characteristic progression from effortful, analytical processing to more integrated, intuitive performance. Novices typically show broader, less focused activation patterns across relevant brain regions, relying heavily on explicit analytical strategies implemented by the central executive network. As expertise develops, there is a shift toward more efficient processing, with greater engagement of the default mode network and enhanced functional connectivity between sensory, semantic, and language regions. This shift reflects the development of what Dreyfus and Dreyfus (1986) termed “intuitive expertise”, i.e., the ability to perceive and respond to complex situations without explicit analysis.

The reciprocal relationship between exemplification and neural plasticity creates what can be described as an “expertise spiral.” As individuals develop greater expertise, they become capable of detecting more subtle properties in exemplars, leading to further refinement of perceptual and conceptual systems. This creates a positive feedback loop where enhanced perceptual capacities enable more sophisticated engagement with exemplars, which in turn drives further perceptual refinement. Expert wine critics, for instance, can detect properties in wines that were imperceptible to them as novices, allowing them to use these wines as exemplars for even more subtle discriminations.

The social dimension of expertise development also plays a crucial role. Communities of practitioners serve as curators of exemplars, selecting and presenting them in ways that scaffold the development of appropriate perceptual and conceptual frameworks. The specialized vocabularies that emerge within these communities both reflect and shape the exemplification practices that drive expertise development. This social scaffolding is supported neurally by the mirror neuron system and other mechanisms of social learning, which allow novices to benefit from the perceptual expertise of more experienced practitioners.

Understanding expertise development through the lens of exemplification and neural plasticity makes it clear how demanding it is the extensive training required by certain domains. As already emphasized, the neural architectures supporting supercomplex experiences do not emerge spontaneously but must be cultivated, and sustained engagement with carefully selected exemplars is a key pillar of this process. This explains why domains like wine tasting, musical performance, and artistic creation typically require years of dedicated practice constantly raising the bar: a progressive refinement of perceptual discrimination, conceptual categorization, and neural organization takes time and effort to develop.

Moreover, this account helps explain individual differences in expertise development. Some individuals may show greater natural sensitivity to domain-relevant properties or more efficient neural plasticity mechanisms, potentially predisposing them to faster skill acquisition. However, the quality and structure of training, and particularly the selection and presentation of exemplars, appears to be at least as important as innate differences in determining expertise outcomes. Who teaches you also makes a huge difference.

The integration of exemplification theory with neuroscientific evidence thus provides a comprehensive account of how individuals develop the capacity for supercomplex experiences. This developmental perspective complements our analysis of the real-time neural mechanisms supporting such experiences, offering a complete picture of how these sophisticated forms of engagement with the world emerge and evolve through the dynamic interplay of perceptual learning, conceptual development, and neural plasticity.

## Supercomplexity and expert cross-modality

The capacity for cross-modal integration and metaphorical mapping represents a distinctive hallmark of expertise in supercomplex domains. While basic cross-modal correspondences appear to be universal, such as the association between high pitch and brightness, experts develop far more sophisticated and stable cross-modal mappings that fundamentally shape both their perception and communication of complex experiences. This section examines how these advanced cross-modal capabilities emerge through expertise and how they contribute to the irreducible nature of supercomplex experiences.

Expert practitioners consistently employ cross-modal metaphors that go beyond simple sensory associations to create rich conceptual frameworks. When wine critics describe tannins as “angular” or “velvety,” musicians characterize tones as “bright” or “dark,” or visual artists speak of “warm” or “cool” colors, they engage neural circuits that were originally evolved for sensorimotor processing. Neuroimaging evidence reveals that exposure to such cross-modal metaphors activates not only language-related regions but also the sensory cortices referenced in the metaphor. When processing tactile metaphors like “a rough day,” subjects show activation in the somatosensory cortex alongside language areas (Lacey et al., 2012). Similarly, action-centered metaphors such as “grasp the idea” recruit motor-related brain regions, demonstrating how the brain repurposes sensorimotor networks for abstract conceptual processing (Desai et al., 2011).

What distinguishes expert cross-modal processing from everyday metaphorical thinking is its systematicity and precision. Computational linguistic analysis reveals that expert descriptions show higher dimensional complexity than novice descriptions while maintaining more consistent internal structure (Wang Q. et al., 2012; Wang X. et al., 2012; Sezille et al., 2014). This suggests that experts develop stable, high-dimensional semantic spaces with characteristic geometric properties that can be reliably applied to new stimuli (Chollet et al., 2005). The stability of these mappings across experts, despite the infinite variety of possible experiences they might describe, indicates that they capture fundamental computational similarities in how different sensory and conceptual domains are organized in expert cognition.

The development of expert cross-modal capabilities appears to involve a bidirectional process. As practitioners develop more sophisticated metaphorical frameworks for describing their experiences, these frameworks in turn shape perceptual discrimination through top-down modulation of sensory processing. Wine experts who consistently use spatial metaphors to describe wine structure show enhanced activation in parietal regions associated with spatial processing when tasting wine. This creates a dynamic feedback loop where conceptual frameworks shape perception, which generates new experiences that further refine the conceptual frameworks.

Cross-modal expertise extends beyond perception and communication to actively shape the design and orchestration of supercomplex experiences. In haute cuisine, expert chefs deliberately manipulate multiple sensory channels to create coherent experiential wholes that transcend simple taste combinations. The integration of visual presentation, aromatic composition, textural elements, ambient sound, and environmental factors reflects sophisticated understanding of how cross-modal correspondences can be leveraged to enhance or transform primary sensory experiences (Spence, 2020; Spence and Youssef, 2019).

For instance, research on restaurant atmospherics demonstrates how environmental factors fundamentally alter flavor perception through cross-modal effects. Changes in lighting color temperature can make wine taste sweeter or more bitter, while background music tempo influences perception of flavor intensity (Motoki et al., 2021; Spence and Carvalho, 2020). Expert chefs and restaurateurs who understand these effects can orchestrate dining experiences where each sensory element reinforces and amplifies the others, creating emergent properties that could not be achieved through food alone.

The neural basis for these sophisticated cross-modal effects involves specialized patterns of connectivity between sensory processing regions and higher-order integration areas. Experts show enhanced functional connectivity between regions processing different sensory modalities, mediated by multisensory integration areas in the superior temporal sulcus and intraparietal cortex. Crucially, these connections are not merely additive but show non-linear interaction effects, where activity in one sensory region modulates processing in another in complex, context-dependent ways.

The temporal dynamics of cross-modal integration in experts also differ from those in novices. While novices tend to process different sensory modalities sequentially before attempting integration, experts show evidence of parallel processing with continuous cross-modal interaction from the earliest stages of perception. This parallel processing enables the rapid, holistic appreciation of complex stimuli that characterizes expert performance in supercomplex domains.

Perhaps most intriguingly, expert cross-modal capabilities appear to involve the development of “meta-modal” representations, i.e., abstract patterns that transcend any single sensory modality. Such meta-modal representations, likely implemented in higher-order association areas, capture structural similarities across different sensory domains. A perfumer’s understanding of “balance” in a fragrance composition, for instance, may share computational properties with a chef’s sense of flavor balance or a visual artist’s perception of compositional equilibrium.

The social transmission of cross-modal expertise presents unique challenges and opportunities. Unlike technical skills that can be explicitly taught, cross-modal mappings often resist direct verbal instruction. Instead, they are typically transmitted through exemplification and guided attention within communities of practice. Master practitioners select and present exemplars that highlight particular cross-modal correspondences, gradually shaping novices’ perceptual frameworks. The specialized vocabularies that emerge within expert communities serve not just to describe these correspondences but to reinforce and stabilize them across practitioners.

Understanding expert cross-modality has important implications for education and training in supercomplex domains. Traditional approaches that treat different sensory modalities in isolation may actually hinder the development of integrated expertise. Instead, training programs that explicitly cultivate cross-modal awareness and provide structured opportunities for exploring sensory correspondences may accelerate expertise development. Some innovative culinary schools, for instance, now include modules on visual design and acoustic properties alongside traditional cooking techniques, recognizing that modern haute cuisine requires integrated multisensory design skills.

The study of cross-modal expertise also reveals fundamental principles about how the brain constructs meaning from sensory experience. The fact that experts across diverse domains independently develop similar metaphorical frameworks suggests that these frameworks might reflect deep structural properties of neural organization rather than arbitrary cultural conventions. This has again implications not just about the nature of expertise but also about that of human cognition more broadly, suggesting that our capacity for abstract thought may be fundamentally grounded in patterns of cross-modal association.

In conclusion, expert cross-modality represents far more than enhanced sensory integration or clever use of metaphor. It reflects a fundamental reorganization of perceptual and conceptual systems that enables practitioners to navigate the irreducible complexity of supercomplex experiences. When developing stable yet flexible frameworks for mapping between sensory and conceptual domains, experts create new possibilities for both experiencing and communicating about phenomena that exceed the descriptive capacity of literal language. This capacity for sophisticated cross-modal integration stands as one of the defining features of human expertise in its most developed forms.

## Neural synchronization in collective supercomplex experiences

While our analysis has primarily examined supercomplex experiences at the individual level, many such experiences, from orchestral performance to ritual ceremonies, are inherently collective. Recent advances in hyperscanning techniques reveal that collective supercomplex experiences involve sophisticated patterns of neural coordination across multiple individuals, adding new dimensions to their complexity and integration.

The phenomenon of inter-brain synchronization during collective activities represents one of the most striking discoveries in recent neuroscience. When musicians perform together, their brains exhibit synchronized patterns of activation that extend far beyond simple motor coordination. Studies using hyperscanning during ensemble performance reveal synchronization in regions associated with prediction, emotional processing, and social cognition (Lindenberger et al., 2009; Müller and Lindenberger, 2019). The degree of this neural synchronization correlates with both objective performance quality and subjective reports of ensemble cohesion, suggesting that collective supercomplex experiences may involve the emergence of temporarily shared neural states across individuals.

This synchronization appears to operate through multiple mechanisms and timescales. At the fastest timescales, millisecond-precision synchronization in motor and auditory regions enables the tight temporal coordination required for ensemble performance. At intermediate timescales, synchronization in regions associated with attention and prediction allows performers to anticipate and adapt to each other’s interpretations. At the slowest timescales, synchronization in regions associated with emotional processing and social cognition may underlie the sense of collective flow or unity that characterizes peak ensemble experiences.

The role of expertise in facilitating inter-brain synchronization reveals important principles about collective supercomplex experiences. Expert performers show more sophisticated patterns of neural coupling than novices, with synchronization occurring in higher-order cognitive regions rather than being limited to basic sensorimotor areas (Novembre and Keller, 2014). This expertise effect extends to asymmetric pairings: when experts perform with novices, they show distinct patterns of neural adaptation, dynamically adjusting their predictive models to accommodate their partner’s limitations while maintaining overall performance quality (Wolf et al., 2018).

Collective ritual practices provide another illuminating window into group supercomplex experiences. Hyperscanning studies of participants in traditional ceremonies reveal synchronized activation in the temporoparietal junction and medial prefrontal cortex, namely regions associated with social cognition and shared intentionality (Konvalinka et al., 2011; Liu et al., 2021). This neural coupling appears to facilitate the collective meaning-making that characterizes ritual experiences, allowing participants to coordinate not just their physical movements but their emotional and cognitive states as well.

The intensity of inter-brain synchronization shows interesting relationships with group size and emotional arousal. Chabin et al. (2022) found that audience brain coupling during live music performances correlates with both crowd size and reported emotional intensity. This suggests that collective supercomplex experiences may involve emergent properties that scale with group participation, potentially explaining why certain experiences such as live concerts, religious ceremonies, sporting events, can achieve qualitatively different impacts in group settings compared to individual consumption, with the size of the crowd playing a major upscaling role.

The mechanisms underlying neural synchronization in groups appear to involve multiple channels of coupling. Shared attention to common stimuli creates basic synchronization through stimulus-locked responses. However, the synchronization observed in collective supercomplex experiences extends far beyond what would be expected from shared stimuli alone. Behavioral coupling through synchronized movements, vocalizations, or even breathing patterns creates additional channels for neural entrainment. The rhythmic aspects of many collective activities, from music to ritual chanting to synchronized movement, may serve to enhance this entrainment through well-established mechanisms of neural oscillatory coupling.

Interestingly, synchronization extends beyond neural measures to include other physiological systems. Studies have documented synchronized heart rate variability, respiratory patterns, and even hormonal fluctuations among participants in collective activities (Ardizzi et al., 2020; Tschacher et al., 2024). This multi-system synchronization suggests that collective supercomplex experiences involve the coordination of entire embodied systems, not just brains, across multiple individuals.

The development of collective expertise involves learning to achieve and maintain appropriate levels of synchronization while preserving individual contribution. Orchestra members must synchronize sufficiently to create cohesive ensemble sound while maintaining enough independence to contribute their unique voice. This balance between synchronization and differentiation appears to be actively regulated, with expert groups showing more flexible and context-appropriate patterns of coupling compared to novice groups (Sun et al., 2020).

The social transmission of supercomplex experiences takes on new dimensions when viewed through the lens of neural synchronization. Master teachers may facilitate learning not just through explicit instruction but by creating states of neural coupling that allow students to directly experience expert patterns of neural activity. This synchronization-based learning may be particularly important for aspects of expertise that resist verbal description, providing a direct neural channel for transmitting embodied knowledge.

Cultural variations in collective supercomplex experiences reveal how different societies have developed distinct technologies for facilitating group neural synchronization. From the polyrhythmic structures of West African drumming to the extended temporal arcs of Indian classical music to the precise synchronization required in Western classical orchestras, different cultural traditions emphasize different aspects of collective neural coordination. These variations suggest that there may be multiple routes to achieving the neural synchronization that underlies collective supercomplex experiences.

The study of neural synchronization also illuminates why certain collective experiences achieve transformative impacts on participants. The temporary dissolution of self-other boundaries that can occur during peak collective experiences may have a literal neural basis in the synchronized activity across individual brains. This synchronization may facilitate not just shared experience but shared learning, allowing insights or states achieved by some group members to propagate through the collective.

Understanding collective aspects of supercomplex experiences has important practical implications. In educational settings, structured group activities that promote neural synchronization might enhance learning of complex skills. In therapeutic contexts, collective activities that facilitate appropriate neural coupling might address conditions involving social cognition deficits. In performance contexts, training methods that explicitly develop synchronization capabilities might enhance ensemble cohesion and collective creativity.

The extension of supercomplex experiences from individual to collective domains reveals new levels of complexity and integration. The neural synchronization observed in group settings suggests that human brains have evolved remarkable, not yet fully understood capabilities for creating temporary super-organismic states where individual neural activities become coordinated into larger functional wholes. These collective states may represent some of the most sophisticated forms of information processing available to human groups, enabling achievements in art, ritual, and collective action, among others, that transcend individual capabilities. Understanding these collective dimensions enriches our framework of supercomplex experiences, revealing how the integration of multiple processing streams can extend beyond individual brains to encompass entire groups engaged in shared meaningful activities.

## Discussion

The framework of supercomplex experiences opens several important theoretical and empirical frontiers in cognitive neuroscience. By identifying a class of experiences that share fundamental characteristics across diverse domains, from wine tasting to musical performance to ritual participation, we highlight organizing principles that transcend traditional disciplinary boundaries. Here we explore the implications of our framework, address potential limitations, and chart directions for future research.

Our analysis reveals that certain domains of human experience operate according to principles that differ qualitatively from those governing simpler forms of cognition. The simultaneous engagement of multiple neural networks, the emergence of irreducible experiential gestalts, and the development of specialized neural architectures through expertise all point toward modes of cognitive operation that standard information-processing models struggle to capture. This suggests the need for new theoretical frameworks that can accommodate the dynamic, integrated nature of sophisticated human capabilities.

The relationship between predictive processing and embodied cognition emerges as a particularly fertile area for theoretical development. While we have drawn primarily on predictive processing frameworks in our neural analyses, we recognize that alternative theoretical perspectives offer complementary insights. The challenge for future work lies not in adjudicating between these frameworks but in developing integrative approaches that can capture how predictive mechanisms, embodied action, and environmental coupling jointly contribute to supercomplex experiences. The skilled intentionality framework proposed by Bruineberg et al. (2018) represents one promising direction, but further work is needed to fully integrate insights from different theoretical traditions.

A critical question concerns the boundaries of supercomplexity. While we have identified five criteria that characterize these experiences, the conceptual thresholds and their measurement remain to be established. Future research should develop operational measures for each criterion, enabling more systematic investigation of which activities qualify as supercomplex and to what degree. This might involve developing behavioral tasks that can distinguish supercomplex from merely complex processing, neuroimaging protocols that can quantify network integration and flexibility, and computational models that can capture the emergent properties of these experiences.

The developmental trajectory of supercomplex capabilities presents another crucial area for investigation. We have examined how expertise develops in adults, but understanding how the capacity for supercomplex experiences emerges through childhood and adolescence could reveal fundamental principles about neural and cognitive development. Do children show early precursors to supercomplex processing in their play and creative activities? How do educational environments support or hinder the development of these capabilities? These questions have important implications for educational practice and child development.

Individual differences in the capacity for supercomplex experiences also warrant systematic investigation. Our framework suggests that variations in network organization, neural plasticity, and cross-modal integration might predispose some individuals toward excellence in particular domains. However, the relative contributions of innate differences versus environmental factors remain unclear. Large-scale studies examining how genetic variation, early experience, epigenetic factors, and training interact to shape supercomplex capabilities could inform both theoretical understanding and practical applications in education and training.

The social and cultural dimensions of supercomplex experiences clearly deserve a deeper exploration. We have examined neural synchronization in collective settings; however, the broader question of how cultures create and transmit frameworks for supercomplex experiences remains understudied. Different societies have developed distinct practices, from tea ceremonies to improvised music traditions to contemplative practices, that cultivate particular forms of supercomplex experience. Comparative studies across cultures would likely reveal both universal principles and cultural variations in how these experiences are structured and transmitted.

The relationship between supercomplex experiences and consciousness itself presents profound theoretical questions. The integration of multiple processing streams, the emergence of irreducible experiential wholes, and the resistance to decomposition that characterize these experiences may offer insights into the neural basis of conscious experience more generally. One might go on to maintain that consciousness itself might be understood as an overarching form of supercomplex experience, emerging from the integration of multiple information streams in ways that create unified phenomenal states. While this remains slightly speculative, the study of supercomplex experiences in expert domains might provide empirical windows into these fundamental questions.

Methodological innovations will be crucial for advancing this research program. Current neuroimaging techniques are powerful but may not fully capture the dynamic, multi-scale processes that characterize supercomplex experiences. Future methods might combine multiple imaging modalities, employ real-time neurofeedback, or develop new analytical approaches that can track information flow across multiple networks simultaneously. The development of more naturalistic experimental paradigms that can preserve the ecological validity of supercomplex experiences, at the same time maintaining experimental control, represents another major methodological frontier.

The implications for artificial intelligence and machine learning deserve further consideration. Current AI systems excel at narrow, well-defined tasks but struggle with the kind of flexible integration that characterizes human supercomplex experiences. Understanding how biological neural networks achieve this integration might inspire new architectures for artificial systems. Conversely, attempts to model supercomplex experiences computationally might reveal principles that are difficult to discern from biological data alone.

Clinical applications represent another important frontier. Many psychiatric and neurological conditions involve disruptions to the neural networks that support supercomplex experiences. Depression, for instance, often involves altered default mode network function and reduced cognitive flexibility. Understanding how supercomplex experiences depend on healthy network dynamics might inform new therapeutic approaches. Activities that deliberately engage multiple networks in integrated ways, from music therapy to mindfulness practices, might be understood and optimized through the lens of supercomplexity.

We must also acknowledge potential limitations and challenges to our framework. The term “supercomplex” might be seen as implying a hierarchy of cognitive experiences, with supercomplex experiences positioned as superior to simpler forms of cognition. This is not our intention. Rather, we aim to identify a particular class of experiences that operate according to distinct principles, without making value judgments about their relative worth. Simple, focused attention has its own importance and beauty, just as supercomplex integration does.

Another challenge concerns the relationship between subjective experience and neural mechanism. We have proposed neural correlates of supercomplex experiences, but the relationship between these objective measures and subjective phenomenology is still to be understood. Future work might employ novel methods for capturing subjective experience, from micro-phenomenology interviews to experience sampling, to better understand how neural dynamics relate to lived experience.

The question of whether supercomplex experiences represent a natural kind or a useful analytical category also deserves consideration. Are we identifying a fundamental distinction in how certain experiences are organized, or are we drawing somewhat arbitrary boundaries in what is actually a continuum? This question has implications for how we understand the evolution of these capabilities and their distribution across species. Do other animals show precursors to supercomplex experiences, or are they uniquely human?

Looking forward, the framework of supercomplex experiences suggests a research program that bridges multiple levels of analysis, from cellular mechanisms to cultural practices, and multiple disciplines, from neuroscience to philosophy to anthropology. Through the identification of common principles across diverse domains of expertise, we hope to contribute to a more integrated understanding of human cognitive capabilities in their most sophisticated forms. The ultimate goal is not just to understand these experiences scientifically but to develop ways of cultivating and sharing them more widely, enriching human experience and expanding the boundaries of what we consider possible.

## Practical applications

The framework of supercomplex experiences suggests practical applications across multiple domains. Each of our five defining criteria (simultaneous engagement of multiple neural networks; specialized neural architectures developed through training; specialized conceptual frameworks; emergent properties from dynamic interactions; and coherent gestalt properties) points toward specific interventions and approaches.

Criterion 1 suggests the value of creating learning environments that deliberately engage multiple cognitive faculties simultaneously. For instance, arts-integrated education could be reconceptualized not just as an enrichment but as a fundamental tool for developing sophisticated predictive models that enhance learning across disciplines. The development of expertise in any field might benefit from deliberately incorporating elements that make experiences supercomplex, such as multiple sensory modalities and creative exploration.

Criterion 2 informs how professional training programs could be enhanced by incorporating insights about the contribution of supercomplex experiences to the shaping of neural architecture. Rather than focusing solely on technical skills, training could be designed to develop the sophisticated integration capabilities that characterize expert performance. This is particularly relevant for fields requiring real-time integration of multiple information streams, such as emergency medicine, air traffic control, or professional sports.

Criterion 3 suggests new approaches to communication and knowledge transfer in professional domains. Understanding how experts develop specialized vocabularies could inform the design of more effective knowledge management systems and training protocols. This is especially relevant for domains where expertise is traditionally difficult to verbalize and transfer.

Criteria 4 and 5 point toward new perspectives on both diagnosis and treatment in clinical settings. Conditions affecting cognitive integration, such as autism spectrum disorders or certain forms of dementia, might be better understood through examining how they impact the processing of supercomplex experiences. Therapeutic approaches might be developed that deliberately engage multiple cognitive faculties through structured supercomplex experiences, potentially enhancing cognitive rehabilitation outcomes.

These practical implications take on different nuances depending on which theoretical framework one adopts. From a predominantly predictive perspective, such educational approaches might be understood as optimizing students’ generative models. From a predominantly enactivist or pragmatist perspective, they might instead be seen as developing students’ capacities for skilled engagement with meaningful environments. The philosophical tensions we outlined earlier thus have practical consequences for how interventions are designed and evaluated.

The insights from studying supercomplex experiences also have important implications for human-computer interaction and interface design. Understanding how humans naturally integrate multiple processing streams could inform the development of more intuitive and effective interfaces. This is particularly relevant for immersive technologies and virtual reality applications, where successful user experience depends on a sophisticated integration of multiple sensory and cognitive streams.

For artificial intelligence development, our framework hints at the importance of developing systems that are capable of handling complex, ambiguous tasks. Current AI systems often excel at narrow, domain-specific tasks but struggle with the kind of flexible integration that characterizes human expertise in supercomplex domains. Understanding how the brain manages supercomplex experiences through dynamic network integration could inspire the design of more sophisticated artificial neural architectures that can handle multidimensional inputs and generate context-sensitive outputs.

Organizational design and management practices could also benefit from applying insights about supercomplex experiences. Realizing how experts develop and maintain the ability to navigate complex, multifaceted situations may be conducive to new approaches to team composition, workplace design, and professional development. This might include creating environments that better support the integration of multiple cognitive streams or developing new approaches to fostering expertise that explicitly acknowledge the role of supercomplex experiences in professional development.

Our framework also has implications for performance optimization in fields ranging from sports to artistic practice. A comprehension of the neural mechanisms that support integrated performance may pave the way to the development of training techniques that enhance coordination between different processing streams. This might include new approaches to mental practice, attention training, and performance preparation that explicitly target the development of integrated processing capabilities.

In the cultural and creative sectors in particular, understanding supercomplex experiences offers valuable insights for both creative practice and active audience involvement. Cultural institutions could design more effective immersive experiences by understanding how multiple sensory and cognitive streams contribute to meaningful engagement with artworks, performances, or exhibitions. For museums and performing arts venues, this might spark the creation of more sophisticated curatorial strategies that deliberately engage multiple processing streams to transform visitor experience and learning. In creative practice, understanding how supercomplex experiences emerge may set the premise to new approaches to artistic training and creative development. This is particularly relevant for interdisciplinary arts practices where success depends on integrating multiple modalities and cognitive frameworks. Furthermore, cultural managers and producers could better design processes and creative environments that support the development of sophisticated integration capabilities among creative professionals.

These practical applications show how theoretical insights about supercomplex experiences can be translated into concrete interventions across multiple domains. However, implementing these applications requires significant attention to context-specific factors and individual differences. Future work should focus on developing and validating specific interventions based on these theoretical insights, with particular attention to how different populations and contexts might require different approaches to developing and supporting supercomplex processing capabilities, with special care for those with a personal and family background of socio-economic deprivation and marginalization.

## Conclusion

The framework of supercomplex experiences may be helpful in advancing our understanding of how the human brain processes and integrates sophisticated multifaceted experiences that resist decomposition into simpler components. Through examination of diverse domains including wine tasting, musical performance, visual art, and culinary creation, among others, we have identified common neural mechanisms that support these experiences while highlighting their distinctive characteristics that set them apart from other forms of complex cognitive processing.

Our analysis draws attention toward three important insights about brain function. First, supercomplex experiences emerge from the coordinated activity of multiple brain networks, particularly through the dynamic interaction of the salience network, default mode network, and central executive network. This coordination enables the simultaneous engagement of sensory processing, emotional evaluation, and cognitive analysis in ways that create emergent properties beyond what would be predicted from examining each component in isolation.

Second, the development of expertise in domains characterized by supercomplex experiences involves significant modifications to neural architecture, from local circuit refinement to large-scale network reorganization. Rather than simply enhancing existing capabilities, expertise development appears to create new forms of integration that support increasingly sophisticated processing of complex stimuli. This suggests that the brain’s capacity for handling supercomplex experiences is itself plastic and can be developed through appropriate training and experience. This could have in turn important implications for future skills building policies in view of the fact that the cultural and creative sectors, where supercomplex experiences possibly abound more than in any other domain, are also those where the development of general-domain skills of literacy, numeracy and problem solving seems to find the best environmental conditions (Sacco et al., 2025).

Third, the role of predictive processing in shaping these experiences extends beyond traditional models of perception and cognition. The brain appears to maintain multiple parallel prediction streams that interact dynamically through reciprocal connections between higher-order association and emotion areas and primary somatosensory and sensorimotor cortices. This sophisticated predictive architecture enables the integration of immediate sensory input with stored knowledge, emotional states, and expectations in ways that support both rapid processing and long-term learning.

Our framework suggests that supercomplex experiences represent a distinct category of neural processing that requires sophisticated integration across multiple domains. This integration is not merely additive but creates emergent properties that characterize expert performance and sophisticated cognitive capabilities. As we look forward to unraveling the neural mechanisms supporting these experiences, we might gain deeper insights into both the fundamental organization of the brain and practical approaches to enhancing human performance across diverse domains.

To this purpose, we need to develop more precise methods for studying the neural dynamics of supercomplex experiences in real-world settings, understanding individual differences in the capacity for handling complexity, and designing targeted interventions to enhance these capabilities. This is challenging work but the promise it holds cannot be ignored.

The framework of supercomplex experiences advances our understanding by identifying a distinct category of experiences characterized by five interconnected criteria. By examining these experiences through multiple theoretical lenses, from predictive processing to enactivism and pragmatism, we gain a more complete picture of how the brain integrates multiple processing streams in sophisticated ways. We have focused primarily on domains like wine tasting, musical performance, and artistic creation, but the framework offers analytical tools that could be applied across many other fields where human expertise manifests. Future research should investigate not only the neural mechanisms supporting these experiences but also their developmental trajectories, cultural variations, and potential applications across educational, clinical, and technological domains. Bridging previously separate research traditions, the concept of supercomplex experiences may open new avenues for shedding new light on some of the most sophisticated capabilities of the human mind.

## Data Availability

The original contributions presented in this study are included in this article/supplementary material, further inquiries can be directed to the corresponding author.

## Footnotes

1
https://shop.klwines.com/products/details/1740376

## Appendix

### A methodological note on predictive processing.

Our framework of supercomplex experiences draws on predictive processing as one of several theoretical approaches to understanding neural function. We acknowledge that the status of predictive processing and Bayesian approaches to cognition remains the subject of ongoing debate in cognitive science and philosophy (Rescorla, 2015; Piccinini, 2020). While some researchers have questioned whether the brain literally implements Bayesian computations, others have raised concerns about the explanatory scope and empirical testability of predictive processing accounts.

Rather than advocating for predictive processing as the singular or definitive account of neural function, we employ it as one useful framework among several for understanding the integration of multiple processing streams in supercomplex experiences. The hierarchical nature of predictive models, with cascading predictions and prediction errors flowing between levels, provides a compelling account of how experts integrate information across multiple timescales and domains. However, our central claims about supercomplex experiences do not stand or fall with the ultimate validity of predictive processing as a comprehensive theory of brain function.

We acknowledge that alternative computational and theoretical frameworks, including traditional information processing accounts, dynamical systems approaches, and enactivist perspectives, offer valuable insights into neural function. Our use of predictive processing terminology should be understood as offering one perspective on the mechanisms supporting supercomplex experiences, not as a commitment to the strongest versions of predictive processing theory that would claim it as the single unifying principle of brain function.

The neural mechanisms we describe, including the coordination between large-scale brain networks, the integration of sensory, semantic, and affective processes, and the expertise-dependent reorganization of neural architectures, are supported by empirical evidence independent of commitments to predictive processing. While we find predictive processing language useful for articulating how these mechanisms might be coordinated, the empirical phenomena themselves can be described in multiple theoretical frameworks.
